# Trend in Cancer Incidence and Mortality in Fukushima From 2008 Through 2015

**DOI:** 10.2188/jea.JE20200202

**Published:** 2021-12-05

**Authors:** Akiko Shibata, Shigehira Saji, Kenji Kamiya, Seiji Yasumura

**Affiliations:** 1 Center for Cancer Control and Information Services, National Cancer Center, Tokyo, Japan; 2 Radiation Medical Science Center for the Fukushima Health Management Survey, Fukushima Medical University, Fukushima, Japan; 3 Department of Medical Oncology, Fukushima Medical University School of Medicine, Fukushima, Japan; 4 Department of Public Health, Fukushima Medical University School of Medicine, Fukushima, Japan; 5 Research Institute for Radiation Biology and Medicine, Hiroshima University, Hiroshima, Japan

**Keywords:** cancer, incidence, population-based cancer registry

## Abstract

**Background:**

Cancer incidence in Fukushima Prefecture, especially thyroid cancer, has been a public concern, since the Tokyo Electric Power Company Fukushima Daiichi Nuclear Power Plants accident following the Great East Japan Earthquake on March 11, 2011; however, cancer incidence for Fukushima residents before and after the accident based on a population-based cancer registry (PBCR) has not been known worldwide.

**Methods:**

We obtained the corrected-incidence data for invasive cancers newly diagnosed from 2008 through 2015 from the Fukushima Cancer Registry. We checked data quality indicators for PBCRs to confirm comparability. We calculated age-standardized annual incidence and mortality of cancer for all-site, thyroid, and leukemia by calendar year and sex, as we did for Tochigi Prefecture and all of Japan as references for comparison. We applied joinpoint trend analysis to test an apparent trend in incidence and mortality.

**Results:**

The corrected incidence data from the Fukushima Cancer Registry had sufficient quality comparable to other PBCRs. For the age-standardized annual incidence by sex and cancer type in Fukushima and Tochigi, we did not detect any joinpoint in trend with statistical significance. Cancer incidence gently increased from 2008 through 2015 nationwide. Incidence and mortality of cancer for Fukushima before the accident was very close to that for Tochigi.

**Conclusions:**

We interpreted the incidence statistics of cancer for Fukushima residents from 2008 through 2015. Our results will provide fundamental statistics for subsequent researchers to assess the relationship between the disaster and cancer incidence among Fukushima residents in the long term.

## INTRODUCTION

Fukushima Prefecture in Japan suffered a major disaster in March 2011. The Tokyo Electric Power Company Fukushima Daiichi Nuclear Power Plants (FDNPS) accident following the Great East Japan Earthquake released radioactive materials into the surrounding area.^1^ While the United Nations Scientific Committee on the Effects of Atomic Radiation (UNSCEAR) reported that radiation exposure from the accident was not high enough to cause an elevated general risk of cancer and other diseases among the general public in 2013, scientific evidence proving this prediction is expected by the public in Japan.^2^

Cancer incidence in Fukushima has become a public concern. A population-based cancer registry (PBCR) for Fukushima residents, called the Fukushima Cancer Registry, was established in December 2010, just before the disaster, and began recording newly diagnosed cancer cases after January 2008. As of August 2013, the registry had not met the quality criteria determined by a cancer surveillance research group supported by the Japanese Ministry of Health, Labour and Welfare (MHLW) for collecting de-identified data of cancer and estimating cancer incidence in 2010 from participating registries.^3^ Furthermore, until enforcement of the Cancer Registration Promotion Act in 2016, cancer was not a reportable disease in Japan. Prefectures, the largest unit of local government in Japan, had organized their cancer registries independently based on their own policy decision, and hospitals and clinics voluntarily reported cancer patients to the PBCRs in accordance with the rules set by each individual prefecture. Therefore, the quality of cancer incidence statistics reported in each PBCR varied until the 2015 incidence year. The background around PBCRs in Japan made it difficult to evaluate any change in cancer incidence for Fukushima residents after the disaster and to compare cancer incidence with that in other prefectures.

**Figure 1.**
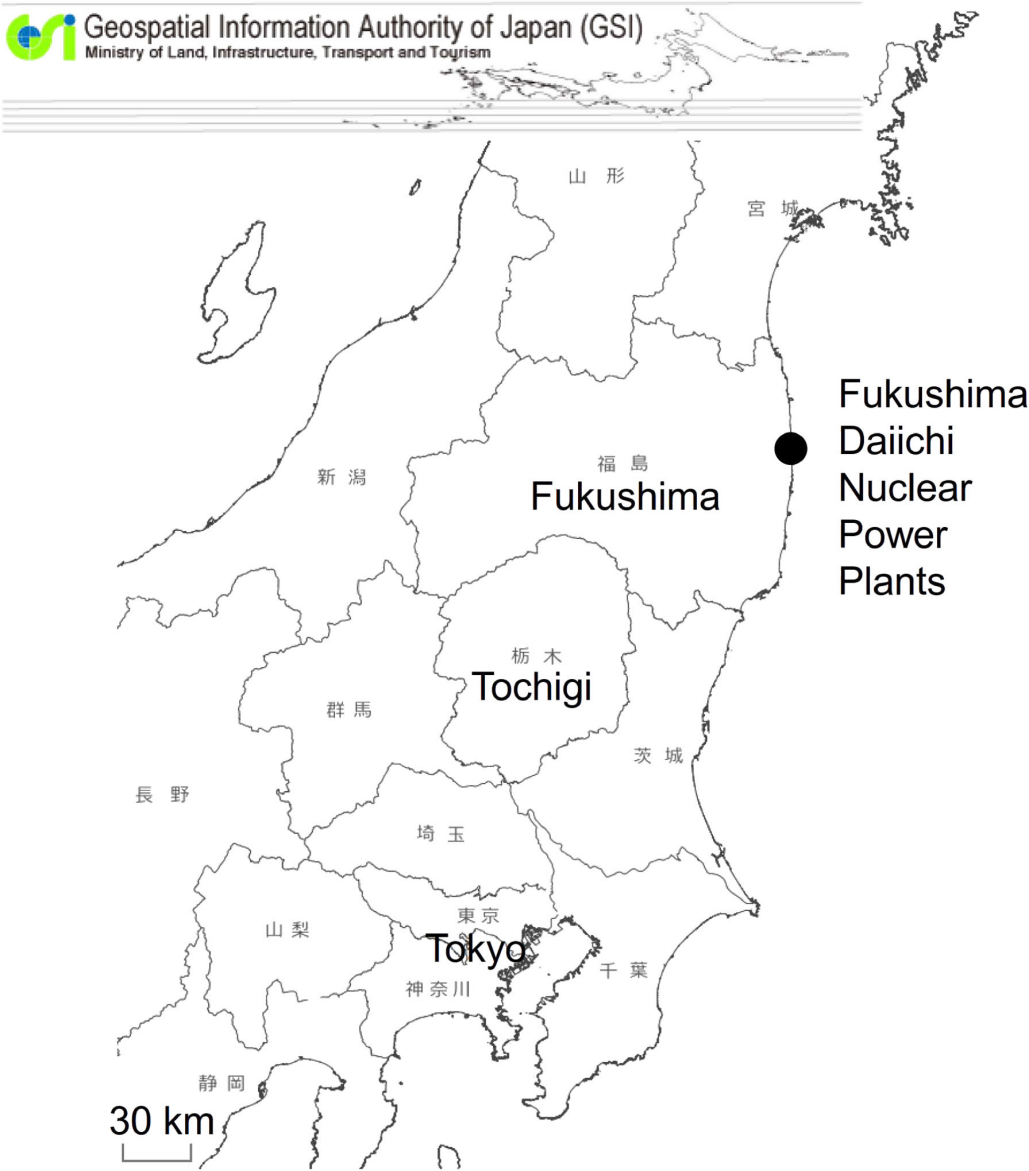
The location of Fukushima Prefecture, Tochigi Prefecture and the Fukushima Daiichi Nuclear Power Plants

In order to find and record un-registered cancer diagnoses from 2008 to 2010 in the Fukushima Cancer Registry database retrospectively, the Fukushima government started collecting cancer incidence reports by hospital-visits in 2013. As a result, the registration quality of the Fukushima Cancer Registry has been improved to meet the criteria set by the research group mentioned above, and the Fukushima government has published Cancer Incidence in Fukushima in 2008–2012 as a prefectural statistical report in March 2017.^4^ As increases in cancer incidence in the Japanese population have been reported over the past several decades,^5^ relative assessment procedures are required to interpret the cancer incidence for Fukushima residents. We tried to solve this problem by referring to cancer incidence in Tochigi Prefecture, located southwest to Fukushima Prefecture (Figure 1). The two prefectures have similar population structure and cancer mortality trends compared to the other prefectures in Japan.^5^ The most important point was that the PBCR of Tochigi was established in 1993 and has been reporting plausible incidence statistics since the 2008 diagnostic year.^6^

In this study, we aimed to make public reliable incidence of cancer in Fukushima before and after the disaster in 2011 through assessing the quality of incidence data derived from the corrected database of the Fukushima Cancer Registry and evaluating trend in incidence and mortality of cancer for all-site, thyroid, and leukemia in 2008–2015.

## METHODS

We used incidence data for invasive cancers diagnosed from January 1, 2008, to December 31, 2015, exported from the Fukushima Cancer Registry database in May 2019, as which represented the corrected incidence for Fukushima residents (Fukushima-corrected) fixed in September 2018. The dataset was fixed as the submission from the Fukushima Cancer Registry to a research group supported by the MHLW that led a project ‘Monitoring of Cancer Incidence Japan (MCIJ) in 2015’.

We referred to cancer incidence and mortality statistics from 2008 through 2015 for Fukushima-first reported, Tochigi, and Japan for comparison. These data were derived from annual reports of MCIJ for each year, as published by the National Cancer Center Japan.^6^ The cancer incidence for the Japanese population in these reports was estimated using data from selected prefectures that satisfied the selection criteria.

We checked several data quality indicators for PBCRs in order to confirm comparability of cancer incidence for Fukushima-first reported, Fukushima-corrected, and Tochigi. We used mortality/incidence ratios (M/I) as an indicator of completeness of the registry, percentage of cases morphologically verified (MV%) as a measure of validity, and percentage of death certification only registrations (DCO%) as a measure of both validity and completeness.^7^

Cancer was registered according to the 3rd edition of the International Classification of Disease for Oncology in the PBCRs in Japan and was further classified based on the 10th edition of the International Classification of Diseases (ICD-10) for Statistics using a common conversion list in Japan. We selected cancer based on the ICD-10 codes in this study; C00–C96 for all-sites, C73 for thyroid, and C91–C96 for leukemia.

We calculated age-standardized annual incidence and mortality using the 1980 Japanese standard population, for all-site, thyroid and leukemia by calendar year and sex. We used joinpoint regression analysis to test whether or not an apparent change in trend was statistically significant and to estimate annual percent change (APC) and average annual percent change (AAPC) which quantified trends in incidence from 2008 through 2015. Joinpoint analyses were done with the Joinpoint Regression Program, Version 4.7.0.0 (Statistical Research and Applications Branch, National Cancer Institute, Bethesda, MD, USA).

The de-identified data of Fukushima-corrected without key table to identify the patients was provided by the Fukushima Cancer Registry. The other incidence data and mortality data used in this article were derived from the MCIJ annual reports and have published or publicly available.

## RESULTS

All quality indicators for the data of Fukushima-corrected between 2008 and 2011 have improved remarkably compared to those of Fukushima-first reported in the past (Table 1). The mean MI ratio for Fukushima-corrected in 2008–2015 was 0.46 (standard deviation [SD], 0.01), was close to that of Tochigi at 0.44 (SD, 0.02). The mean MV% for Fukushima-corrected showed a 3.8% increase in 2008–2015 and the standard deviation became smaller compared than the mean MV% of Fukushima-first reported, it improved slightly higher than that of Tochigi. The DCO% for Fukushima-corrected became lower than that for Fukushima-first reported and for Tochigi, and kept good quality throughout the 8 years.

**Table 1.** MI ratio, MV%, and DCO% from 2008 to 2015

	2008	2009	2010	2011	2012	2013	2014	2015	mean	(SD)
MI ratios										
Fukushima-first reported	0.63	0.57	0.53	0.54	0.46	0.45	0.44	0.44	0.51	(0.07)
Fukushima-corrected	0.47	0.47	0.46	0.47	0.44	0.44	0.44	0.45	0.46	(0.01)
Tochigi	0.46	0.45	0.45	0.45	0.45	0.44	0.44	0.41	0.44	(0.02)
MV%										
Fukushima-first reported	77.8	74.4	74.3	81.4	84.4	84.5	84.9	83.7	80.7	(4.5)
Fukushima-corrected	85.3	83.9	84.7	84.1	84.9	84.5	84.9	83.7	84.5	(0.6)
Tochigi	78.1	79.1	79.3	80.2	81.1	82.1	82.5	85.3	81.0	(2.3)
DCO%										
Fukushima-first reported	16.7	19.4	20.7	3.8	1.8	1.8	1.8	1.6	8.5	(8.8)
Fukushima-corrected	2.4	2.7	2.4	2.1	1.5	1.6	1.8	1.6	2.0	(0.5)
Tochigi	13.5	12.8	12.2	9.3	9.0	8.7	8.0	3.5	9.6	(3.2)

DCO%, the percentage of death certificate only cases; MI, mortality incidence; MV%, the percentage of cases morphologically verified; SD, standard deviation.

For the age-standardized annual incidence by sex and cancer type in Fukushima and Tochigi, we did not detect any joinpoint in trend from 2008 through 2015 with statistical significance (Table 2). For overall and men in Japan, the incidence of all-site increased, with APC of 2.7% (95% confidence interval [CI], 0.6–4.8%) and 2.2% (95% CI, 0.1–4.4%) from 2008 to 2011, respectively; however, the trend after 2011 was not statistically significant (eTable 1). The observed trend was robust even after excluding thyroid cancer for overall, but not for men. Age-standardized annual incidence of all-site cancer of Fukushima-corrected for both men and women were very close to those for Tochigi, and were consistently lower than those of Japan. Incidence of all-site cancer in Japan gently increased from 2008 through 2015, as did Fukushima-corrected and all-site incidence in Tochigi. The AAPC for overall from 2008 to 2015 was 0.8% (95% CI, 0.1–1.6%) for Fukushima, 1% (95% CI, 0.7–1.3%) for Tochigi and 1.2% (95% CI, 0.5–2.0%) for Japan, respectively. For the incidence of all-site excluding thyroid cancer for Fukushima-corrected decreased from 0.8% to 0.6% (95% CI, −0.2 to 1.3%), but this was not statistically significant. Those for Tochigi and Japan were 0.9% (95% CI, 0.6–1.2%) and 1.1% (95% CI, 0.4–1.8%) and were statistically significant.

**Table 2.** Age-standardized annual incidence between 2008 and 2015 by sex and cancer type (per 100,000 population, 1985 Japanese population model), AAPC, and 95% CI

	2008	2009	2010	2011	2012	2013	2014	2015	Joinpoint	AAPC	95% CI
All-site											
Overall											
Fukushima-corrected	333.1	331.4	346.5	333.8	355.1	349.9	348.0	351.2	0	0.8	0.1 to 1.6
Tochigi	327.4	331.8	334.4	336.0	338.3	345.9	342.7	353.8	0	1.0	0.7 to 1.3
Japan	337.5	342.7	351.5	366.0	365.8	361.9	362.1	369.1	1	1.2	0.5 to 2.0
Men											
Fukushima-corrected	417.7	418.0	438.3	423.1	443.4	425.9	427.8	429.0	0	0.3	−0.5 to 1.1
Tochigi	409.7	408.7	419.8	416.1	415.7	420.3	412.8	428.1	0	0.4	0.0 to 0.9
Japan	421.5	425.1	433.0	449.0	447.8	436.1	433.0	434.8	1	0.5	−0.3 to 1.2
Women											
Fukushima-corrected	271.4	269.0	280.2	268.0	290.1	296.0	289.1	294.2	0	1.4	0.5 to 2.4
Tochigi	267.1	277.6	271.7	277.0	280.3	291.5	292.2	298.9	0	1.5	1.1 to 2.0
Japan	275.9	282.6	292.6	305.5	305.0	307.8	310.8	322.6	0	2.1	1.5 to 2.6

All-site excluding C73											
Overall											
Fukushima-corrected	326.1	324.6	339.5	327.3	346.4	337.8	337.0	338.5	0	0.6	−0.2 to 1.3
Tochigi	322.0	325.4	328.2	328.7	331.6	339.0	335.7	346.4	0	0.9	0.6 to 1.2
Japan	330.5	334.9	343.5	357.7	357.5	352.7	352.7	360.0	1	1.2	0.4 to 2.0
Men											
Fukushima-corrected	414.3	414.5	434.2	419.6	439.3	419.0	421.0	422.1	0	0.2	−0.7 to 1.0
Tochigi	406.5	404.6	416.4	412.6	412.0	417.1	409.4	423.8	0	0.4	0.0 to 0.9
Japan	417.8	420.9	428.5	444.8	443.7	431.1	428.1	429.8	1	0.4	−0.3 to 1.2
Women											
Fukushima-corrected	260.6	258.8	270.3	258.5	276.8	278.7	274.0	275.7	0	1.0	0.2 to 1.8
Tochigi	259.5	268.7	262.6	265.8	270.6	280.7	281.6	288.4	0	1.4	0.9 to 2.0
Japan	265.6	271.4	281.2	293.2	292.6	294.5	296.9	309.5	0	2.0	1.4 to 2.6

Thyroid											
Overall											
Fukushima-corrected	7.0	6.8	6.9	6.5	8.7	12.1	11.0	12.7	0	10.8	5.4 to 16.5
Tochigi	5.4	6.4	6.2	7.3	6.7	6.9	7.0	7.4	0	3.2	0.7 to 5.8
Japan	7.0	7.8	8.0	8.3	8.3	9.2	9.4	9.1	0	3.7	2.3 to 5.2
Men											
Fukushima-corrected	3.4	3.4	4.1	3.5	4.1	6.9	6.8	6.9	0	13.2	6.9 to 19.8
Tochigi	3.2	4.1	3.4	3.5	3.7	3.2	3.4	4.3	0	1.4	−2.9 to 5.9
Japan	3.7	4.2	4.5	4.2	4.1	5.0	4.8	4.9	0	3.6	1.1 to 6.2
Women											
Fukushima-corrected	10.7	10.2	9.9	9.5	13.2	17.3	15.2	18.5	0	9.9	4.3 to 15.8
Tochigi	7.6	8.8	9.1	11.3	9.7	10.8	10.6	10.5	0	4.0	0.5 to 7.6
Japan	10.3	11.2	11.5	12.3	12.3	13.3	13.9	13.1	0	3.8	2.4 to 5.3

Leukemia											
Overall											
Fukushima-corrected	6.0	4.9	5.9	5.4	5.5	6.9	6.4	4.9	0	0.6	−4.2 to 5.7
Tochigi	6.1	5.1	6.7	6.9	6.1	6.0	7.2	6.2	0	1.6	−2.5 to 5.8
Japan	6.1	6.2	6.2	6.6	6.5	6.3	6.3	6.5	0	0.7	−0.2 to 1.5
Men											
Fukushima-corrected	7.4	6.0	7.6	7.1	7.3	9.0	8.1	5.6	0	0.4	−5.5 to 6.7
Tochigi	8.3	7.1	8.3	8.8	7.6	7.0	8.9	6.6	0	−1.0	−5.4 to 3.6
Japan	7.6	7.8	7.6	8.0	8.2	7.7	8.0	7.9	0	0.5	−0.5 to 1.4
Women											
Fukushima-corrected	4.8	4.0	4.5	3.9	4.0	4.9	4.9	4.4	0	0.9	−2.8 to 4.8
Tochigi	4.3	3.3	5.3	5.1	4.6	5.1	5.6	5.9	0	5.5	0.6 to 10.6
Japan	4.8	4.8	5.0	5.3	5.0	5.0	4.8	5.3	0	0.7	−0.8 to 2.3

AAPC, average annual percent change; CI, confidence interval.

For the incidence of thyroid cancer in overall, the AAPC was 10.8% (95% CI, 5.4–16.5%) for Fukushima-corrected in contrast to 3.2% (95% CI, 0.7–5.8%) for Tochigi and 3.7% (95% CI, 2.3–5.2) for Japan, respectively. By sex, the AAPC of thyroid cancer for men in Fukushima-corrected was 3.3% larger than that for women in Fukushima. Regarding the incidence of leukemia was almost level off, a statistically significant positive AAPC was observed only for women in Tochigi, and those for the other population were shown level off trends.

Age-standardized annual mortality of all-site cancer of Fukushima was similar to those for Tochigi and Japan overall (Table 3). The mortality of all-site cancer for Fukushima, Tochigi and Japan, except for men in Tochigi, gradually decreased from 2008 through 2015 without a joinpoint. The AAPC for overall was −1.4% (95% CI, −1.6 to −1.2%) for Japan, −1.1% (95% CI, −1.6 to −0.6%) for Fukushima and −1.3% (95% CI, −1.9 to −0.6%) for Tochigi, respectively. For men in Tochigi, the APC was −0.5% (95% CI, −2.0 to 1.1%) from 2008 to 2012 and −2.8% (95% CI, −5.1 to −0.4%) from 2012 through 2015 (eTable 2).

**Table 3.** Age-standardized annual morality between 2008 and 2015 by sex and cancer type (per 100,000 population, 1985 Japanese population model), AAPC, and 95% CI

	2008	2009	2010	2011	2012	2013	2014	2015	Joinpoint	AAPC	95% CI
All-site											
Overall											
Fukushima	129.3	128.8	128.9	125.9	126.5	121.8	119.5	121.9	0	−1.1	−1.6 to −0.6
Tochigi	133.4	130.1	130.9	130.3	129.0	126.3	126.1	118.9	0	−1.3	−1.9 to −0.6
Japan	135.0	131.5	130.8	129.5	127.2	125.6	123.8	121.3	0	−1.4	−1.6 to −1.2
Men											
Fukushima	184.2	180.2	182.6	179.2	179.5	171.1	164.0	167.8	0	−1.5	−2.3 to −0.8
Tochigi	182.5	181.4	179.6	180.8	179.8	171.2	171.1	164.1	1	−1.4	−2.3 to −0.6
Japan	189.0	183.3	182.4	179.4	175.7	172.5	168.9	165.3	0	−1.8	−2.0 to −1.6
Women											
Fukushima	88.8	91.0	89.2	86.1	86.0	85.4	87.0	87.7	0	−0.5	−1.2 to 0.2
Tochigi	96.6	91.7	93.7	91.8	89.2	92.0	91.4	84.4	0	−1.2	−2.2 to −0.2
Japan	94.2	92.2	92.2	91.8	90.3	89.7	89.4	87.7	0	−0.9	−1.1 to −0.7

Thyroid											
Overall											
Fukushima	0.4	0.4	0.4	0.4	0.7	0.4	0.6	0.4	0	3.1	−8.4 to 16.0
Tochigi	0.6	0.7	0.4	0.4	0.3	0.5	0.6	0.5	0	−1.9	−11.3 to 8.5
Japan	0.5	0.5	0.6	0.5	0.5	0.5	0.5	0.5	1	−0.9	−2.1 to 0.3
Men											
Fukushima	0.3	0.3	0.4	0.5	0.9	0.3	0.3	0.3	1	−1.3	−7.2 to 4.9
Tochigi	0.2	0.7	0.3	0.4	0.3	0.3	0.4	0.5	0	−0.9	−14.9 to 15.5
Japan	0.4	0.5	0.5	0.5	0.4	0.4	0.4	0.4	0	−0.9	−2.6 to 0.7
Women											
Fukushima	0.5	0.4	0.5	0.3	0.6	0.4	0.9	0.4	0	6.2	−6.8 to 21.0
Tochigi	0.8	0.7	0.4	0.4	0.3	0.6	0.6	0.5	0	−4.2	−14.1 to 6.8
Japan	0.6	0.6	0.6	0.6	0.6	0.6	0.6	0.5	1	−1.5	−2.0 to −1.0

Leukemia											
Overall											
Fukushima	3.1	3.0	3.5	2.8	2.8	3.6	2.3	3.1	0	−1.1	−6.3 to 4.3
Tochigi	3.2	2.9	3.3	2.8	3.1	2.8	3.7	3.1	0	0.8	−2.9 to 4.7
Japan	3.5	3.5	3.5	3.4	3.2	3.3	3.2	3.3	0	−1.3	−2.0 to −0.6
Men											
Fukushima	4.1	4.4	4.9	4.1	4.1	5.0	2.9	4.0	0	−1.8	−7.5 to 4.2
Tochigi	4.0	4.2	4.1	3.5	4.4	3.6	5.1	4.1	0	1.5	−3.2 to 6.4
Japan	4.7	4.7	4.7	4.5	4.4	4.3	4.3	4.4	0	−1.4	−2.1 to −0.7
Women											
Fukushima	2.4	2.1	2.2	1.8	1.7	2.4	1.9	2.4	0	−0.5	−5.9 to 5.2
Tochigi	2.5	1.8	2.7	2.3	2.0	2.0	2.3	2.4	0	−0.6	−5.5 to 4.6
Japan	2.5	2.5	2.5	2.5	2.3	2.4	2.3	2.4	0	−1.2	−2.4 to 0.0

AAPC, average annual percent change; CI, confidence interval.

With respect to leukemia and thyroid cancer, significant increases or decreases in mortality from 2008 through 2015 were not seen in either Fukushima or Tochigi, except for thyroid cancer for women in Japan and leukemia for men in Japan. Regarding the mortality of thyroid cancer, a joinpoint of trend was detected for overall in Japan, for men in Fukushima, and women in Japan. For men in Fukushima, the mortality increased with APC of 30.9% (95% CI, 17.6–45.6%) from 2008 to 2012 and decreased with APC of 32.3% (95% CI, −43.7 to −18.6%) from 2012 through 2015. The AAPC from 2008 through 2015 was −1.3% (95% CI, −7.2 to 4.9%).

We showed crude incidence rates in Fukushima from 2008 through 2015 by site, sex, and 5-year age group in the eTable 3.

## DISCUSSION

UNSCEAR noted that their statement phrase that there was “no discernible increase (of cancer incidence)” in Fukushima after the FDNPS accident did not rule out the possibility of future excess cancer cases.^2^ Where risk for stochastic effects would be sufficiently large in an exposed population of sufficiently large size, compared to the normal statistical variability in the baseline incidence of cancer in the population, an increased risk due to irradiation may be discernible in the cancer statistics. Therefore, the baseline incidence of cancer in Fukushima residents would be fundamental information to evaluate excess risk of cancer due to the disaster in March 2011.

In spite of this, the Fukushima Cancer Registry has assumed a guarded stance toward scientific publication of cancer incidence for several reasons. First, they knew that cancer incidence would be underestimated for several years from the start of the operation of their PBCR. Cancer incidence for Fukushima in 2008–2010 was considered unstable and unreliable because in December 2010, the Fukushima Cancer Registry started to collect cancer cases diagnosed after January 2008, retrospectively. Second, they had difficulties specific to Japan in evaluating the completeness of cancer registration. Unexpected or implausible trends in cancer incidence are used as a potential manifestation of changes in completeness of registration.^8^ The concept is extended to includes comparisons of results with those observed in other populations that might have been expected to manifest similar cancer incidence rates.^8^ Namely, since cancer incidence derived from other populations may reflect specific local variations in the prevalence of risk factors, or the presence or intensity of screening for some cancers and registration quality, comparisons should only be made between populations with similar characteristics and comparable data quality. However, it was difficult to find PBCRs in Japan covered population with similar characteristics and comparable data quality before improving the data quality of the Fukushima Cancer Registry. Third, cancer incidence in Fukushima could be affected by structural changes in population due to evacuation in and after 2011. The number of evacuees outside Fukushima was approximately 63,000 in January 2012, accounting for 3% of the population before the disaster.^9^

We confirmed the corrected incidence data of cancer from 2008 through 2015 for Fukushima residents. We observed that the completeness of registration improved dramatically from 2008 to 2011 as a result of collecting incidence reports based on hospital-visits since 2013. The quality of Fukushima-corrected data became comparable to the standard data quality for monitoring of cancer incidence in Japan.^7^^,^^8^ Therefore, we thought that we could now provide convincing baseline data of cancer incidence in Fukushima before the disaster on March 2011. In addition, we found that Tochigi Prefecture, located southwest of Fukushima Prefecture, had a similar population structure and cancer incidence and mortality trends from 2008 through 2015, for a huge increased AAPC of incidence for thyroid cancer in Fukushima-corrected. It could be inferred that general risks factors of cancer, such as lifestyle and infection, would be similar in both Fukushima and Tochigi before the disaster, and the cancer incidence in Tochigi would be a useful reference indicator when evaluating excess risk of cancer in Fukushima.

The incidence of thyroid cancer was shown a significant increased trend in Fukushima-corrected, Tochigi, and Japan; however, the magnitude of AAPC in Fukushima-corrected was clearly different from those in Tochigi and Japan. Describing the incidence trend of thyroid cancer for Fukushima in detail, it seemed that the sudden increase was observed in 2013, the trend became gentle since 2014. The apparent trend was not detected as a joinpoint. In Fukushima, the sensitive ultrasound-based thyroid screening of those aged 18 years or younger and living in Fukushima Prefecture at the time of the accident has been started since October 2011 by the Fukushima Health Management Survey (FHMS). The first-round screening was done for 300,000 attendees until March 2014 (later, extended to April 2015).^10^ Approximately 13% and 60% of the attendees was examined until March of 2012 and March of 2013, respectively. It has been predicted that a large number of thyroid cysts and solid nodules, including a number of thyroid cancers “that would not normally have been detected without such intensive screening” would be detected by the screening.^11^ We confirmed that more than 80% of thyroid cancers in persons aged under 25 years in Fukushima from 2008 through 2015 were cases after 2012, in contrast to about 40–70% of those over 25 years old (eTable 3). It would make sense that thyroid cancer diagnosed in thyroid screening attendees aged 21 or younger at the time of the screening accounted for an unnatural increase in incidence in Fukushima in 2013.

Concerning change in the population structure of Fukushima, the population was approximately 2,029,000 in 2010 and 1,914,000 in 2015.^12^ The number of evacuees outside Fukushima had decreased by two-thirds in 2015.^9^ A decrease in total population due to a declining birth rate and an aging population are social issues in Japan, as well as in Fukushima. Observed changes on the proportions of total Japanese population of three major age groups (under 15 years old, 15 to 64 years old, and 65 years and older) between 2010 and 2015, showed the population aged 15–64 years old and the population aged under 15 years old in Fukushima had a steeper declining tendency compared to other prefectures.^12^ Therefore, part of the possible population at risk might have been eliminated from a denominator for the calculation of cancer incidence in Fukushima since 2011. This means that the cancer incidence of Fukushima residents would be underestimated when presuming maximum cancer risk in the future due to the disaster. Meanwhile, the number of evacuees from Fukushima to Tochigi has been stable around 2,500–3,000 since 2011.^9^ The stability for the Tochigi population was another reason considering trends in cancer incidence for Tochigi before and after the disaster would be good reference data for that of Fukushima.

We believe that the excess cancer incidence due to the disaster could be evaluated using a cohort of the FHMS managed by the Fukushima Prefectural government.^1^ The subjects were all residents of Fukushima Prefecture as of March 2011, and their long-term health is being monitored. As for checking the incidence of cancer, the Fukushima Prefectural government has the privilege of using the National Cancer Registry in Japan, which contains data for registered cancer diagnoses since 2016 based on the Cancer Registration Promotion Act. Unfortunately, since the National Cancer Registry cannot provide data on cancer diagnoses before 2015, it was important that the completeness of the Fukushima Cancer Registry was substantialized to provide a survey of cancer information in Fukushima. UNSCEAR recommended in the Fukushima 2017 White Paper that linkage of outcome information—including mortality and incidence of thyroid cancer, other cancer, and non-cancer diseases; birth defects; and clinical and laboratory findings—with radiation exposure and information on age, sex, and other risk factors, would permit the most informed assessment of health experience and risk; this would maximize the capability to address important questions that both scientists and the public may have.^10^ The linkage between high quality population-based cancer registry data and the FHMS would contribute to these research needs.

Before concluding this article, we would like to describe a point to be aware of when analyzing and interpreting cancer incidence in Japan. We evaluated trends in cancer incidence up to 2015 because we observed an unnatural dramatic increase in cancer incidence in 2016 in Japan.^13^ The sudden increase in cancer incidence for the 2016 year could be caused by hospital reporting cancer cases all at once for the 2016 year in response to enforcement of the Cancer Registration Promotion Act. When studying cancer incidence and trends using data derived from PBCRs in Japan, researchers should consider some artificial variations in cancer incidence following methodological changes in PBCRs.^7^^,^^8^ The shortness of available period for trend analysis is a limitation of our study. At least seven data points should be observed in order to consider allowing a joinpoint, and the default maximum number of joinpoints for eight data points is determined “1”. The shortness of the available period also influences the study power of joinpoint analysis. Zanetti et al indicated that detecting statistically significant trends in population based incidence or mortality rates depended on the size of their covered population, the levels of incidence rates, and duration of trend period and type of temporal variation.^14^ According to their model, a low mortality rate, such as that of thyroid cancer, among a small population like Fukushima or Tochigi, with about 2 million population, would be unlikely to detect a statistically significant trend with a power of 80%. If the population covered is not large enough for detecting the investigated effect, we should consider pooling data with a prolonged analytic period. In the case of incidence rates, we could merge data from other cancer registries with homogenous registration methods and comparable data quality.^14^

In conclusion, our results will provide fundamental statistics for subsequent researchers to assess the relationship between the disaster on March 2011 and cancer incidence among Fukushima residents in the long term.

## APPENDIX A. SUPPLEMENTARY DATA

eTable 1. Results of joinpoint regression analysis on the trends in incidence (2008–2015)

eTable 2. Results of joinpoint regression analysis on the trends in mortality (2008–2015)

eTable 3. Crude incidence rate in Fukushima between 2008 and 2015 by site, sex, and 5-year age group
